# Lumbar radiculopathy due to foraminal stenosis with ossification of the ligamentum flavum: a case report

**DOI:** 10.1093/jscr/rjab405

**Published:** 2021-09-23

**Authors:** Shuta Ushio

**Affiliations:** Department of Orthopaedic Surgery, Kudanzaka Hospital, Chiyoda-ku, Tokyo, Japan

## Abstract

The patient was an 82-year-old woman with a diagnosis of lumbar radiculopathy due to foraminal stenosis accompanied by ossification of the ligamentum flavum (OLF). Computed tomography scans of the lumbar spine revealed ossification in the capsular portion of the ligamentum flavum around the L2–L3 facet joint. In addition, computed tomography images acquired a few months before the onset of radiculopathy had shown that the ossification site had gradually expanded to include the superior articular process. The patient’s symptoms disappeared immediately after excision of the OLF. Histopathological examination of the resected specimen indicated replacement of degenerated ligamentum flavum with ossified tissue via a gradual endochondral ossification process. It is important to be aware that foraminal stenosis can in rare cases occur due to OLF, even in the lumbar spine.

## INTRODUCTION

Ossification of the ligamentum flavum (OLF), also known as ossification of the yellow ligament (OYL), is generated from mature lamellar bone and associated with proliferating cartilage that replaces the ligament flavum (i.e. endochondral ossification) [1]. OLF is known to occur predominantly in the Asian population [1–4]. Several studies that used computed tomography (CT), which might contribute to a higher detection rate, have found a high prevalence of OLF in the range of 36–64% [5–7].

OLF typically involves the lower third of the thoracic spine [3, 5, 8]. The non-central type of OLF in particular occurs mainly in the lower thoracic region [6]. OLF is a clinically important cause of spinal cord compression and compressive myelopathy [1]. However, radiculopathy caused by lumbar foraminal stenosis due to OLF has not been reported in the literature. Here, we describe a case of radiculopathy secondary to OLF in the lumbar spine that required surgery.

## CASE REPORT

**Figure 1:**
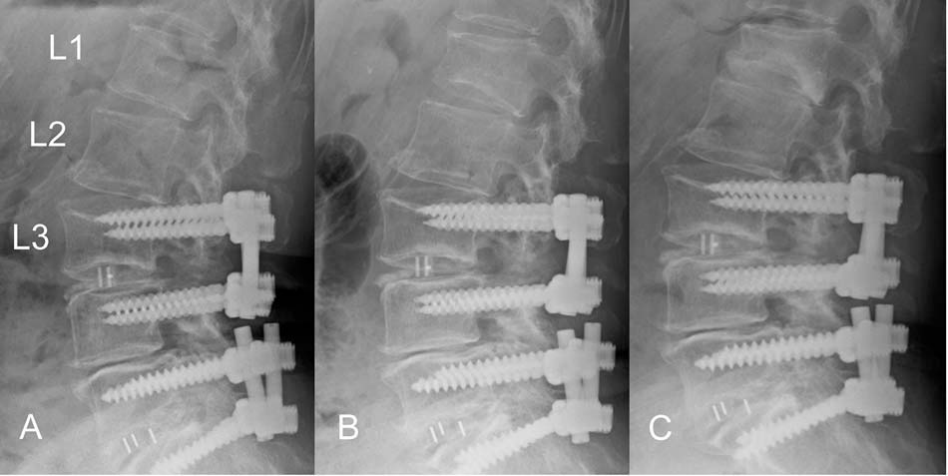
Time course of a vertebral fracture at L1. (**A**) Radiographic image obtained just before the vertebral fracture occurred. (**B**) The vertebral fracture had occurred at L1 4 months before surgery. (**C**) Radiographic image obtained when the patient presented with symptoms of radiculopathy shows no apparent posterior slip of the L2 vertebra. The L1 vertebral body was deformed into a wedge shape.

**Figure 2:**
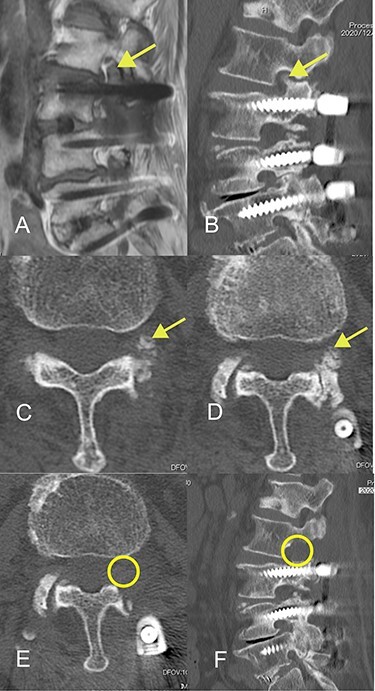
Magnetic resonance and computed tomography (CT) images acquired at the time of onset of radiculopathy. (**A**) Sagittal T1-weighted magnetic resonance image showing moderate foraminal stenosis at the L2–L3 level. (**B**) Sagittal CT image showing ossification of the ligamentum flavum protruding into the foramen. (**C**, **D**) Axial CT image showing OLF in the capsular portion. (**E**, **F**) Postoperative CT image confirming adequate removal of the ossification of the ligamentum flavum.

The patient was an 82-year-old Japanese woman who had presented 2 months earlier with pain and numbness in her left lower extremity. She had a history of vertebral fracture at L1 4 months previously for which she had been treated with a lumbar orthosis for 2 months (Fig. 1). Magnetic resonance imaging of the lumbar spine demonstrated foraminal stenosis at the L2–L3 level (Fig. 2A). CT images of the lumbar spine revealed ossification in the capsular portion of the ligamentum flavum around the L2–L3 facet joint (Fig. 2B–D). She underwent L2–L3 foraminotomy with excision of the capsular portion of the OLF. The patient’s symptoms disappeared immediately after surgery. Postoperative CT scans showed good decompression at the operated level (Fig. 2E and F).

**Figure 3:**
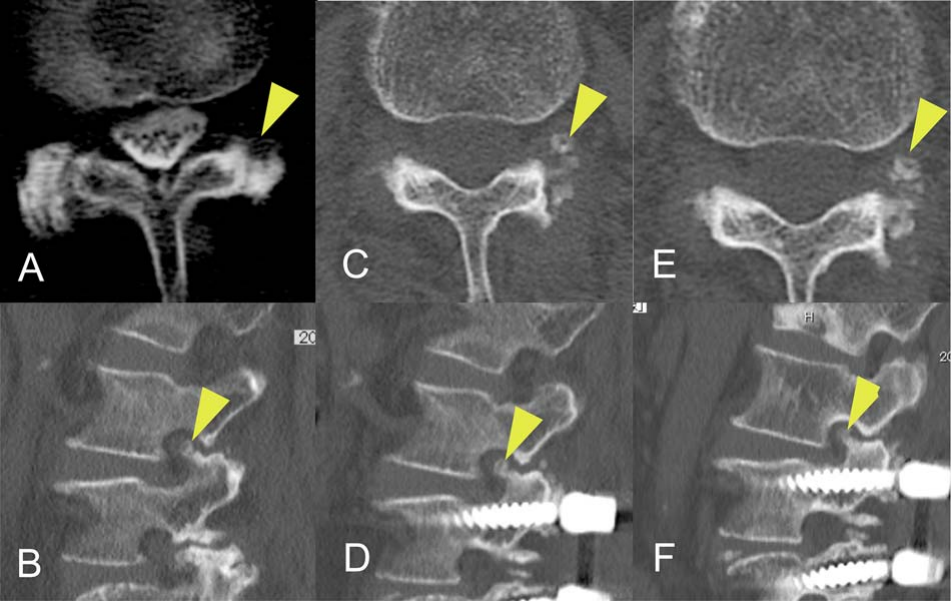
Progression of ossification of the ligamentum flavum (OLF) on computed tomography (CT) images obtained before onset of radiculopathy. (**A**) OLF was not found on a CT scan obtained 8 years earlier. (**B**) A CT image obtained 20 months earlier shows a small isolated area of OLF in the foramen. (**C**, **D**) A CT image acquired 4 months earlier shows that the area of OLF had grown slightly and was integrated with the superior articular process. (**E**, **F**) A CT image obtained at the time of surgery shows further expansion of OLF.

CT images acquired a few months before the onset of radiculopathy had shown a small isolated area of ossification, suggesting that the ossification site had gradually expanded to include the superior articular process over a period of months (Fig. 3).

**Figure 4:**
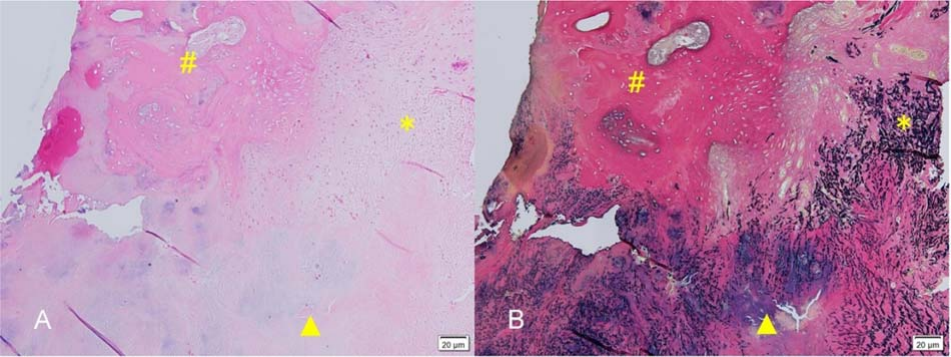
(**A**) Hematoxylin and eosin-stained micrograph showing chondrocytes near the area of ossification (hash). Formation of bone in hyaline cartilage is indicated by osteoblasts (triangle). There was no cartilage cap, as would be found in osteochondroma. (**B**) Elastica van Gieson-stained micrograph showing sparse and degenerated elastic fibers in the ligamentum flavum.

Histopathological examination of the resected specimen showed a degenerated ligamentum flavum and a distinctive ossified plaque within the ligamentum flavum. Chondrocytes were identified near the area of ossification but there was no cartilage cap to suggest osteochondroma. These findings indicated that the degenerated ligamentum flavum had been gradually replaced by ossified tissue via an endochondral ossification process (Fig. 4) and were considered sufficient to warrant a diagnosis of OLF.

## DISCUSSION

Foraminal stenosis is usually caused by overhang of the superior articular processes, thickening of the ligamentum flavum or disc bulging but very rarely by OLF, which typically causes immobilization of the facet joint. However, in this case, OLF occurred without immobilization of the facet joint and was the likely cause of radiculopathy.

The ligament flavum consists of a capsular portion and an interlaminar portion, and most cases of OLF arise at the enthesis of the capsular portion. Although the pathogenesis of OLF is still unclear, it is thought to involve both endogenous and mechanical factors. Endogenous factors include increased expression of bone morphogenetic protein-2, transforming growth factor-beta and vascular endothelial growth factor [9, 10]. Mechanical stress on the ligamentum flavum, particularly its capsular portion, is also thought to be a main contributor to development and aggravation of OLF [11, 12] and usually occurs at the lower thoracic level. The strong traction applied to the ligamentum flavum in a lower thoracic spine with kyphosis has been suggested to affect the ossification mechanism [13]. Mori *et al.* reported that the non-central type of OLF occurs predominantly in the lower thoracic region whereas the central type of OLF is more likely to occur in the upper to midthoracic region [6]. They also hypothesized that an altered mechanical environment may affect the distribution of the different types of OLF. Therefore, if the mechanical stress on the ligament flavum at the lumbar level is the same as that in the lower thoracic region, it would not be surprising if OLF was generated in the capsular portion even in the lumbar spine. In our patient, it is possible that a slight change in the alignment of the thoracolumbar junction due to the vertebral body fracture at L1 accompanied by increased stress on the ligamentum flavum at L2–L3 contributed to the formation of OLF.

The changes in CT findings over time in this case (Fig. 3) confirm that the ossification was initially confined to the ligamentum flavum but gradually expanded to the point where it constricted the nerve root at the foramen. However, our review of the literature did not yield any reports of OLF as a cause of radiculopathy at the foramen. Furthermore, although there are some reports on the classification of OLF, most did not include an isolated type. Sato *et al.* proposed that thoracic OLF be classified as lateral, extended, enlarged, fused or tuberous [14]. Mori *et al.* subsequently recommended a modified classification system based on whether OLF was small, medium, large, extra-large or central-type [6]. However, Saiki *et al.* identified four types of OLY, namely, spiny, placoid, nodular and isolated [15]. They also identified that 1% were of an isolated free type, which they defined as a form of ossification that is free in the ligamentum flavum and has no continuity with the articular processes. We consider that our case could be classified as the isolated free type of OLF originally described by Saiki *et al.* and suspect that this rare type of OLF may have been overlooked in more recent studies, particularly those using CT images. Furthermore, the radiographic and CT images obtained before the onset of radiculopathy in this patient were valuable in that we were able to confirm progression of OLF before the onset of symptoms.

Awareness that OLF may occur in isolation in the intervertebral foramen and cause foraminal stenosis even at the lumbar spine level may lead to identification of more patients with the same pathology as our case.
